# Clustering of energy balance-related behaviors in 5-year-old children: Lifestyle patterns and their longitudinal association with weight status development in early childhood

**DOI:** 10.1186/1479-5868-9-77

**Published:** 2012-06-21

**Authors:** Jessica S Gubbels, Stef PJ Kremers, Annette Stafleu, R Alexandra Goldbohm, Nanne K de Vries, Carel Thijs

**Affiliations:** 1 Department of Health Promotion, Maastricht University, PO Box 616, 6200 MD, Maastricht, the Netherlands; 2 NUTRIM School for Nutrition, Toxicology and Metabolism, Maastricht University, PO Box 616, 6200 MD, Maastricht, the Netherlands; 3 TNO, PO Box 360, 3700 AJ, Zeist, the Netherlands; 4 TNO, PO Box 2215, 2301 CE, Leiden, the Netherlands; 5 School for Public Health and Primary Care (CAPHRI), Maastricht University, PO Box 616, 6200 MD, Maastricht, the Netherlands; 6 Department of Epidemiology, Maastricht University, PO Box 616, 6200 MD, Maastricht, the Netherlands

**Keywords:** BMI, Diet, Eating pattern, Factor analysis, Overweight, Physical activity, Principal component analysis, Screen-based behavior, Sedentary behavior

## Abstract

**Background:**

This study identified lifestyle patterns by examining the clustering of eating routines (e.g. eating together as a family, having the television on during meals, duration of meals) and various activity-related behaviors (i.e. physical activity (PA) and sedentary screen-based behavior) in 5-year-old children, as well as the longitudinal association of these patterns with weight status (BMI and overweight) development up to age 8.

**Methods:**

Data originated from the KOALA Birth Cohort Study (N = 2074 at age 5). Principal component analysis (PCA) was used to identify lifestyle patterns. Backward regression analyses were used to examine the association of lifestyle patterns with parent and child background characteristics, as well as the longitudinal associations between the patterns and weight status development.

**Results:**

Four lifestyle patterns emerged from the PCA: a ‘Television–Snacking’ pattern, a ‘Sports–Computer’ pattern, a ‘Traditional Family’ pattern, and a “Fast’ Food’ pattern. Child gender and parental educational level, working hours and body mass index were significantly associated with the scores for the patterns. The Television–Snacking pattern was positively associated with BMI (standardized regression coefficient *β* = 0.05; *p* < 0.05), and children with this pattern showed a positive tendency toward being overweight at age 8 (Odds ratio (OR) = 1.27, *p* = 0.06). In addition, the Sports–Computer pattern was significantly positively associated with an increased risk of becoming overweight at age 7 (OR = 1.28, *p* < 0.05).

**Conclusions:**

The current study showed the added value of including eating routines in cross-behavioral clustering analyses. The findings indicate that future interventions to prevent childhood overweight should address eating routines and activity/inactivity simultaneously, using the synergy between clustered behaviors (e.g. between television viewing and snacking).

## Background

Various studies have examined the co-occurrence, or ‘clustering’, of ‘energy balance-related behaviors’ (i.e. diet- and activity-related behaviors) in children (see e.g. [1-9]). Clusters are combinations of behaviors which are more prevalent than would be expected from the prevalence of the individual behaviors [10]. The potential synergy between energy balance-related behaviors could be used in obesity prevention interventions, by applying an integrated approach, for example by addressing multiple behaviors simultaneously [11].

Identifying the clustering of behavioral patterns in young children is important, since dietary and physical activity (PA) habits are formed in the early life stages and track into later life [12,13], even into adulthood [14,15], so adult lifestyle is often already established during childhood. Principal component analyses (PCA) and cluster analyses are both frequently used and validated methods to examine the existence of behavioral patterns [16,17], and the patterns they reveal are comparable [18]. Several studies, including our own previous study in the same study sample as that reported on in the current paper [9], have applied one of these two techniques to examine cross-behavioral energy balance-related clustering (i.e. clustering between PA and dietary intake) in children. Examples of patterns that have often been reported are a ‘sedentary–snacking’ pattern, in which intake of unhealthy food items clusters with sedentary screen-based behavior (i.e. television and computer use [1-7,9]); and an ‘all-round-healthy’ pattern of healthy food intake and high levels of PA [2-5,7,8] and/or low levels of screen-based behavior [3,7]. However, all these studies used measures of *dietary intake* in their clustering analyses, i.e. focused on *what* was eaten by the children. They did not include information on *how* and *where* these items were consumed, i.e. a child’s *eating routines*. Examining the clustering of such eating routines would provide information regarding the context in which the clustered energy balance-related behaviors occur.

A few studies did include some eating routines in the cross-behavioral examination of lifestyle patterns in children, mostly in addition to various dietary intake behaviors. Kontogianni et al. [19] recently reported a pattern involving a high breakfast consumption and a high eating frequency in children, in combination with a Mediterranean diet, which was negatively associated with body mass index (BMI). They did not find any cross-behavioral clustering (i.e., clustering across eating routines and activity-related behavior). Another study included television viewing during dinner in its analysis, in addition to television viewing in general, PC use and PA [20], and found some indications of cross-behavioral clustering: having the television on during dinner clustered negatively with PA and positively with television viewing in general. Children with this pattern had higher cross-sectional odds of being overweight. A third study that incorporated eating routines in its examination of clustering found that eating fast food clustered with screen-based behavior, whereas breakfast and dinner frequency each clustered with dietary intake behaviors (e.g. vegetable intake) but not with other eating routines or activity-related behaviors [4].

None of the three studies incorporating eating routines described above included a longitudinal follow-up for weight status [4,19,20], and their cross-sectional findings with regard to weight status thus remain purely indicative. Furthermore, none of these studies systematically included a wide range of eating routines [4,19,20]. This led us to the question which eating routines are important for children’s weight development and health, and could thus be meaningfully included in clustering analyses. Various reviews have examined eating routines [21-25]. Unhealthy dietary intakes (e.g. high fat intake, low fruit and vegetable intake) and increases in body mass index (BMI) have been linked to having the television on during meals (19, 20), and eating out [22]. By contrast, reviews have linked healthy dietary intake patterns and a healthy weight status with eating together as a family at the table [21-23,25] and with a high breakfast frequency [23,24]. In addition to the negative effects of eating out, the habits of eating take-out food at home and eating fast food or snacks in general have also been found to be associated with unfavorable intake patterns and weigh development (e.g. [26,27]). Finally, shorter meal duration has been found to be associated with obesity (e.g. [28]).

Not only have there been no studies that systematically examined clustering of eating routines instead of dietary intake behaviors, but almost all previous cross-behavioral clustering studies assessed activity-related behaviors using only one or two measures to summarize all activity-related behaviors (e.g. minutes of exercise [4]), instead of differentiating between various types of physical activity and sedentary behavior. The only exceptions we know of are a study that differentiated between moderate and vigorous PA [8], and our previous study in the current study population [9].

The primary aim of the current study was to examine clustering between eating routines and various distinct activity-related behaviors in 5-year-olds. We thereby sought to establish whether eating routines cluster with activity-related behaviors, in addition to the clustering between dietary intake and activity-related behaviors which has been repeatedly shown before. Our secondary aim was to examine the association between the patterns identified and the characteristics of the children and their parents, in order to be able to predict which children will show which behavioral patterns. This may help to identify high-risk groups that could be targeted by preventive interventions. Finally, we examined the association between such lifestyle patterns and longitudinal weight status development up to age 7–8 years, to ascertain which behavioral patterns are obesogenic, and which patterns protect against overweight development.

## Methods

### Respondents and procedure

The KOALA Birth Cohort Study is a prospective cohort study in the Netherlands which started in the year 2000. Healthy pregnant women were recruited from an existing cohort assembled for a study on pregnancy-related pelvic girdle pain, as well as through ‘alternative lifestyle’ recruitment channels (e.g. anthroposophist midwives and organic food shops [29]). The latter group (17.9%) could have an alternative lifestyle with regard to dietary habits (e.g. eating organic food), child rearing, vaccination schemes or antibiotics use. All participating parents signed informed consent, and ethical approval was obtained from the Maastricht University/University Hospital Maastricht medical ethics committee. A total of 2834 women completed questionnaires during pregnancy and after birth.

### Questionnaires

When the children reached the age of about 5 years (mean age = 60.2 months; SD = 6.5), parents (i.e. either the mother *or* the father) completed a questionnaire on various background characteristics, their children’s eating routines, activity-related behaviors, BMI, and other relevant factors (N = 2074, 73.2%). Longitudinal follow-up questionnaires assessing the child’s BMI were sent around ages 6–7 (mean age = 77.9 months, SD = 7.4; N = 2002, 96.5%) and 7–8 years (mean age = 86.2 months, SD = 7.8 months; N = 1828, 88.1%). There was no selective attrition with regard to children’s or paternal variables between the assessment at age 5 and the follow-up at age 7–8 years (p > 0.05). Mothers who dropped out were, however, heavier than those not dropping out (BMI 24.4 vs. 23.7 kg/m², respectively; p < 0.01), and were more likely to have a low educational level (10.7% vs. 6.9%; p < 0.01). The questionnaires are described in more detail below.

#### Children’s activity-related behavior

Children’s activity-related behavior was assessed using the Standard Questionnaire for measuring physical activity, which is used in Dutch Youth Health Care [30]. Parents were asked on how many days in a normal week during the last 4 weeks their child had gone to school on foot or by bicycle, played sports at school (e.g. during physical education lessons), played sports at a sports club, and/or played outside (outside school hours). Response options ranged from ‘Never or less than one day a week’ to ‘7 days a week’). In addition, the average duration of these activities on such a day was assessed. The response options were ‘Less than half an hour a day’, ‘Half an hour to 1 hour a day’, ‘1-2 hours a day’, ‘2-3 hours a day’ and ‘3 hours or more a day’. The duration in minutes and the number of days were multiplied to calculate the total number of minutes spent on each activity per week. For all response options that comprised a certain range, the midpoint value of the response option was used for further calculations (e.g. ‘1-2 hours a day’ was recoded as 90 minutes a day). Sedentary screen-based behavior was assessed in a similar manner, asking for television watching (including videos and DVDs) and computer playing. Table 1 provides an overview of the activity-related behaviors assessed.

**Table 1:** Energy balance-related behavior and descriptives (N = 2074)

**Behavior**	**Content**	**mean**	**sd**
** *Activity-related behavior* ***(minutes/week)*			
Active means of transport	*Walking or cycling to school*	45.9	54.6
School sports	*Sports at school, including physical education and swimming lessons as part of the curriculum*	83.7	62.3
Sports at a club	*Sports at a sports club (e.g. swimming, soccer, ballet)*	43.4	53.1
Playing outside	*Playing outside, excluding school recess*	622.9	367.5
Watching television	*Television watching, including videos and DVDs*	357.8	248.2
Computer use	*Using the computer, including using the internet and playing games on a regular or games computer*	53.4	103.0
** *Eating routines* **			
Meal frequency *(per week)*	*Total number of breakfasts, lunches and dinners consumed per week*	20.8	0.7
Take-out meal frequency *(per week)*	*Total number of meals consumed from a take-out or at a restaurant per week*	0.5	0.5
Snack frequency *(per week)*	*Total number of snacks consumed per week*	22.4	10.2
Duration of meals *(minutes)*	*Average number of minutes a meal takes*	30.6	7.7
Eating together *(%)*	*Percentage of meals consumed together with at least one other family member*	90.6	11.1
Eating at the table *(%)*	*Percentage of meals consumed at the dinner table*	89.8	15.4
Eating with television on *(%)*	*Percentage of meals consumed while television was on*	11.1	17.7

#### Children’s eating routines

Based on previous research (e.g. [21-28]), various eating routines were taken into account (see Table 1). We assessed the number of meals consumed by a child by asking ‘On how many days a week did your child eat breakfast, lunch or dinner?’. We also asked ‘How many times a week do you eat meals together as a family or with part of the family (at least one parent and the participating child)?’; ‘How many times a week does your child have his/her meals at the dinner table?’; and ‘How often is the television on during meals?’. Response options for these questions ranged from 0 to 7 days a week. In addition, we asked parents how long an average meal of the child lasted. Response options were ‘0-15 minutes’, ’15-30 mins’, ’30-45 mins’, ‘45-60 mins’ and ‘>60 mins’. Parents had to answer all these questions for breakfast, lunch and dinner separately. We also asked ‘How often during the past four weeks did your child eat at a restaurant or snack bar or eat take-out food from a restaurant or snack bar at home?’. Response options were ‘Never’, ‘Once a month’, ‘2-3 times a month’, ‘Once a week’, ‘2-3 times a week’, ‘4-5 times a week’ and ‘6-7 times a week’. The final question assessed how often the child ate or drank anything between the main meals (excluding water). This question had to be answered for each of the following possible snacking moments: ‘between breakfast and lunch’; ‘between lunch and dinner’; ‘between dinner and going to sleep’; ‘during the night’. Response options ranged from ‘Never’ to ‘Four times or more a day’. Just as with the activity-related behaviors, we used the midpoint value of all response options that comprised a certain range for further calculations.

Frequency of meals, take-out meals and snacks were calculated as weekly totals for the three meals (breakfast, lunch and dinner). Duration of meals was calculated as the average for all the meals, in minutes. Eating together, eating at the table, and eating with the television on were all calculated as an average percentage of the total number of meals.

#### Children’s BMI and weight status

Parents were asked to report their child’s weight and height around ages 5, 6–7 and 7–8 (measured without shoes and clothes, specified to one decimal), to allow the child’s body mass index (BMI, i.e. weight (kg) / (height (m))²) to be calculated. BMI was then recoded into age- and gender-specific BMI z-scores compared to the national reference population [31]. A BMI z-score ≥85th percentile was considered to indicate overweight [32].

#### Child and parental background factors

The child’s gender and birth weight were assessed as background variables. In addition, various parental background factors were assessed, including the number of hours a week that fathers and mothers worked, their highest completed education and their country of birth. Educational level was recoded into three levels (low, medium, high [33]). Country of birth was recoded as ‘Netherlands’ versus ‘other’. Parents were also asked to indicate their own weight and height, which we used to calculate parental BMI (in kg/m²).

### Analyses

The analyses were conducted using SPSS 15.0. P-values <0.05 were considered statistically significant. PCA with oblique rotation was performed to examine behavioral patterns. Oblique rotation is the most appropriate method when the factors from the PCA are expected to intercorrelate [34], as was the case in the current study. All variables were standardized, and a cut-off of an eigenvalue >1 was used to determine the number of components [34]. Behavioral items with absolute component loadings >0.4 were considered part of the pattern [35]. Behavioral pattern scores were calculated using the regression method [34]. As our primary research aim was the examination of clustering, all children with complete data at age 5 (N = 2074) were retained in these analyses.

Backward linear regression analyses were used to examine the association between various background characteristics and the pattern scores. The characteristics included in the analyses were child gender, birth weight and BMI z-score at age 5, and maternal and paternal working hours, educational level, country of birth and BMI. Dummy variables for high and low compared to medium educational level were created for the regression analyses. The analyses were further adjusted for recruitment group (alternative versus conventional).

Separate backward linear and logistic regression anal\yses were conducted to examine the association of each of the pattern scores with children’s BMI and weight status (i.e. being normal weight or overweight) at the follow-up moments at age 6–7 and 7–8, corrected for BMI or weight status at age 5. As these analyses were corrected for BMI z-score or weight status at age 5, the outcomes reflect longitudinal development of BMI and weight status between ages 5 and 6–7, and between ages 5 and 7–8, respectively. These analyses were further adjusted for the background characteristics described above, and included all children with complete follow-up data for BMI (N = 2002 and N = 1828, respectively).

## Results

An overview of the background characteristics of the children and their mothers and fathers can be found in Table 2.

**Table 2:** Background characteristics of the study population (N = 2074)

			**Mean ± sd**	**Prevalence (%)**
**Child**	Gender	male		51.3%
		female		48.7%
	Birth weight	*(grams)*	3524 ±508	
	BMI z-score	5 y	−0.27 ±0.99	
		6-7 y	−0.29 ±0.94	
		7-8 y	−0.32 ±0.90	
**Mother**	Employment	*(hours per week)*	17.9 ±11.1	
	Educational level	Low		8.0%
		Medium		37.8%
		High		54.2%
	Country of birth	Netherlands		97.0%
		other		3.0%
	BMI	*(kg/m*^*2*^*)*	24.0 ±3.8	
	Alternative lifestyle^a^	*(yes vs. no)*		17.9%
**Father**	Employment	*(hours per week)*	37.8 ±10.1	
	Educational level	Low		12.8%
		Medium		33.8%
		High		53.4%
	Country of birth	Netherlands		96.3%
		other		3.7%
	BMI	*(kg/m*^*2*^*)*	25.0 ±3.1	

^a^*Recruited through alternative channels vs. through conventional channels.*

### Child behavior

Table 1 provides an overview of the children’s activities and eating routines. Of the activities assessed, most time was spent on playing outside: almost 1.5 hours a day (623 minutes a week) on average. Other PA behaviors were performed far less. The computer was used by the children for an average of less than one hour a week (53 minutes/week), but the relatively large standard deviation (SD = 103) reveals that there was a skewed distribution, with 49.6% of the children not using the computer at all, and other children using it for up to 3.5 hours a day. The total average time spent being physically active was 795 minutes/week (SD = 378). The total screen viewing time (i.e. television and computer use) was 411 minutes/week (SD = 293).

On average, children consumed 3 meals a day (20.8 per week), with about one meal per two weeks being a take-out meal or eating out. Meals took approximately half an hour on average. In addition, the children consumed over 3 snacks a day (22.4 per week). Most meals were eaten together with a family member and at the table. The television was on during 11.1% of the meals.

### Clustering of child behavior: lifestyle patterns

The PCA revealed four patterns; the component loadings of each behavior for each of these patterns are listed in Table 3. The first pattern, which we called the ‘Television–Snacking’ pattern, included television watching, a high snacking frequency, eating with the television on, and not eating at the table. The second pattern included using an active means of transport, playing sports at school and at a sports club, plus computer use. This pattern was named the ‘Sports–Computer’ pattern. The third pattern showed a cross-behavioral clustering of active means of transport with the number of meals and eating together as a family, and was labeled the ‘Traditional Family’ pattern. A fourth pattern included a high intake of take-out meals and a short duration of meals, so literally “Fast’ food eaters’. These four patterns explained over 44% of the variance in the original variables. Playing outside was the only variable that was not included in any of the patterns, but it had more or less equal absolute component loadings for all four patterns, slightly below the cut-off value of 0.4.

**Table 3:** Component loadings of principal component analysis on energy balance-related behavior

	**Pattern**			
	**1**	**2**	**3**	**4**
** *Activity-related behavior* **^a^				
Active means of transport	0.045	**0.428**	**0.478**	−0.341
School sports	−0.045	**0.552**	−0.154	−0.223
Sports at a club	0.012	**0.650**	0.167	0.045
Playing outside	0.361	−0.309	0.322	−0.390
Watching television	**0.425**	0.386	−0.060	0.246
Computer use	0.081	**0.458**	−0.023	0.344
** *Eating routine* **				
Meal frequency^b^	−0.182	−0.059	**0.476**	0.227
Take-out meal frequency^b^	0.074	−0.046	0.089	**0.497**
Snacks frequency^b^	**0.491**	−0.098	0.231	0.064
Duration of meals^c^	−0.104	−0.004	−0.008	**−0.640**
Eating together^d^	−0.008	0.041	**0.696**	−0.003
Eating at the table^d^	**−0.717**	−0.018	0.334	−0.013
Eating with television on^d^	**0.782**	0.115	−0.150	0.097
**Variance Explained** (%)^e^	17.2	9.9	9.2	7.9

Notes: Results of oblique principal component analysis. Bold component loadings are absolute loadings >0.400 and are thus considered part of the pattern.

Pattern 1: Television–Snacking pattern.

Pattern 2: Sports–Computer pattern.

Pattern 3: Traditional Family pattern.

Pattern 4: ‘Fast’ Food pattern.

^a^ Minutes per week.

^b^ Number per week.

^c^ Minutes per meal.

^d^ Percentage of total number of meals.

^e^ Total variance in the behavioral variables explained by the 4 patterns is 44.1%.

### Associations between background characteristics and lifestyle patterns

Various child and parental background characteristics were related to the patterns (Table 4). Girls had lower scores for (i.e. were less likely to exhibit) the Television–Snacking pattern and the ‘Fast’ Food pattern. Both mothers’ and fathers’ educational level was inversely related to the Television–Snacking pattern. In addition, both high and low paternal educational levels were related to lower scores for the Sports–Computer pattern, while high paternal educational level was negatively related to the Traditional Family pattern. Mothers who worked more hours had children with lower scores for both the Sports–Computer pattern and the Traditional Family pattern. Finally, maternal BMI was positively related to scores for the Television–Snacking pattern, while paternal BMI was positively related to the Sports–Computer pattern.

**Table 4:** Association of child and parental background variables with pattern scores

			**Standardized regression coefficient ( *β* )**			
			**Pattern 1**	**Pattern 2**	**Pattern 3**	**Pattern 4**
**Child**	Gender	*(female vs. male)*	−0.04*	^a^	^a^	−0.05*
**Maternal**	Educational level	*(high vs. medium)*	−0.11***	^a^	^a^	^a^
		*(low vs. medium)*	0.14***	^a^	^a^	^a^
	Work	*(hours per week)*	^a^	−0.07**	−0.20***	^a^
	BMI	*(kg/m²)*	0.07**	^a^	^a^	^a^
**Paternal**	Educational level	*(high vs. medium)*	−0.11***	−0.05*	−0.07**	^a^
		*(low vs. medium)*	0.03	−0.06*	0.00	^a^
	BMI	*(kg/m²)*	^a^	0.06**	^a^	^a^

Notes: BMI, body mass index. Results of backward regression analyses with pattern scores as dependent variable. All analyses were adjusted for recruitment group (alternative vs. conventional lifestyle).

Variables excluded from all four final models were birth weight and BMI z-score of the child; mother’s country of birth; father’s working hours and country of birth.

Pattern 1: Television–Snacking pattern.

Pattern 2: Sports–Computer pattern.

Pattern 3: Traditional Family pattern.

Pattern 4: ‘Fast’ Food pattern.

^a^ Variable not included in the final model.

* *p* < 0.05; ** *p* < 0.01; *** *p* < 0.001.

### Association of lifestyle patterns with longitudinal BMI and weight status development

Table 5 shows the associations of the patterns with longitudinal BMI and weight status development. The Television–Snacking pattern was positively associated with an increased BMI z-score at age 7–8 (standardized regression coefficient *β* = 0.05, *p* < 0.05), and children with this pattern showed a tendency toward being overweight at age 7–8 (Odds ratio (OR) = 1.27, *p* = 0.06). In addition, the Sports–Computer pattern was significantly associated with an increased overweight risk at age 6–7 (OR = 1.28, *p* < 0.05). The Traditional Family and ‘Fast’ Food patterns were not related to the longitudinal BMI and weight status development.

**Table 5:** Association of pattern scores with longitudinal BMI and weight status development

	**BMI z-score**		**Overweight**	
	***Standardized β (95%CI)***		***Odds ratio (95%CI)***	
	**6-7 years**	**7-8 years**	**6-7 years**	**7-8 years**
**Pattern 1**	^a^	0.05*	^a^	1.27†
		(0.00-0.09)		(0.99-1.65)
**Pattern 2**	^a^	^a^	1.28*	^a^
			(1.03-1.60)	
**Pattern 3**	^a^	^a^	^a^	^a^
**Pattern 4**	^a^	^a^	^a^	^a^

Notes: BMI, body mass index; CI, confidence interval. Results of backward linear and logistic regression analyses with BMI z-score and overweight as dependent variables, respectively. The analyses were corrected for BMI z-score/weight status at age 5, and thus reflect development of BMI and weight status between ages 5 and 6–7, and between ages 5 and 7–8, respectively. All analyses were further adjusted for recruitment group; child gender and birth weight; and parental educational level, working hours, country of birth and BMI.

Pattern 1: Television–Snacking pattern.

Pattern 2: Sports–Computer pattern.

Pattern 3: Traditional Family pattern.

Pattern 4: ‘Fast’ Food pattern.

^a^ Variable not included in the final model.

^†^*p* < 0.10; * *p* < 0.05; ** *p* < 0.01; *** *p* < 0.001.

## Discussion

This study examined the clustering of activity-related behaviors and eating routines among young children, and is the first to include a literature-based range of eating routines. The fact that all these eating routines clustered with activity-related behaviors and/or other eating routines shows the value of moving beyond interpreting someone’s diet as merely what that person consumes. As such, our study shows the importance of incorporating the context of these behaviors, in order to establish a more informative typology of children with high scores for a particular pattern. At a methodological level, the inclusion of eating routines instead of dietary intake increases the compatibility of these two behavioral categories, since activity-related behavior measures also tend to include context (e.g. differentiating between sports at school and at a sports club). In addition, this study was the first to examine the longitudinal association between these patterns and weight status development.

We identified four behavioral patterns, two of which were cross-behavioral (i.e. covering eating routines as well as activity-related behaviors), while the third only covered eating routines and the fourth only activity-related behaviors. The first cross-behavioral pattern was named the ‘Television–Snacking’ pattern. Children with high scores for this pattern watch much television, often eat with the television on, have a high snacking frequency, and are more likely not to eat at the table. This pattern is similar to a pattern found in older children (9–14 years old) by Te Velde and colleagues [20], who also found that having the television on during dinner clustered with television viewing in general, as well as less PA. However, they did not include any eating routines other than having the television on during dinner. In addition to the study by Te Velde and colleagues and our current study, numerous clustering studies incorporating dietary intake instead of eating routines have reported similar associations between television use and snacking [1-7,9]. The association between television viewing or having the television on while eating on the one hand and snacking on the other has previously been attributed to various mechanisms, including the idea that sedentary activities offer a context that promotes passive snacking [36], the stimulating influence of snack commercials [37], and the distracting influence of watching TV while eating, disrupting the habituation to food cues (e.g. satiety) [38]. High scores for the Television–Snacking pattern were found to be associated with higher odds of being overweight and having a higher BMI at follow-up. This longitudinal association extends the findings of cross-sectional studies [2,20], and is in line with previous findings in the current cohort [9].

A second cross-behavioral pattern we found was what we named the ‘Traditional Family’ pattern. Children with high scores for this pattern frequently use active means of transport, do not skip meals, and often eat together with the family. To our knowledge, such a pattern has not been identified previously, and we think it reflects a typical traditional Dutch family lifestyle. Dutch families traditionally eat all meals together: even during lunch, children often return home to eat together with their family (or part of the family), which is possible because primary schools are generally at walking (or at most cycling) distance from home (average distance: 700 m [39]). Eating together implies transport (which may include using active means) and could thus provide an explanation for the association between using active means of transport and eating as a family. The Dutch are well-known for their bicycle use, having the highest level of bicycle use in Europe [40]. Viewing this pattern as a traditional lifestyle pattern also fits in with the finding that maternal working hours were negatively associated with the scores for this pattern.

The ‘Sports–Computer’ pattern comprises both high computer use and high levels of PA resulting from the use of active means of transport, engaging in school sports and playing sports at a sports club. This cluster might be explained by the competitive element involved in both sports and computer games, which appeals to certain children, but further research would be needed to confirm this hypothesis. A similar pattern has previously been found in a study by Jago et al. [41], who examined activity-related behavior patterns in 10- and 11-year-olds. In their study, the group of children having a so-called high active–high sedentary pattern accumulated the highest mean number of minutes of moderate to vigorous PA, even higher than the children in the high activity–low sedentary group. The study by Jago et al. [41] and our current study therefore both stress that it is important to consider PA behaviors as well as sedentary behaviors when evaluating a child’s activity-related behavior (e.g. for intervention purposes). The current study revealed an increased overweight risk at age 6–7 for children with high scores for this pattern, which could indicate that the Sports–Computer pattern may be problematic in young children. In line with this, a growing body of evidence shows that sitting time might be more predictive of weight status and health than time spent being physically active (see e.g. [42-44]). This underlines the importance of interventions focusing on reducing sedentary time, in addition to promoting physical activity. The fact that television viewing and computer use clustered within different patterns shows the importance of assessing these behaviors separately, and not as one measure of sedentary screen-based behavior. Another reason to assess screen-based behaviors separately is that previous research has reported television use in youngsters to be negatively, not positively, associated with other sedentary behaviors, including computer use [45].

The fourth pattern found in the current study was a pattern combining a high frequency of consuming take-out meals or eating out with a short average duration of meals. Children with high scores for this pattern were thus literally ‘fast’ food eaters. This pattern was not related to weight status at follow-up, possibly as a result of the low frequency of this behavior, as consuming take-out meals or eating out occurred, on average, only once every 2 weeks in the whole study population.

Various background characteristics proved to be related to the pattern scores. Boys had higher scores for the Television–Snacking pattern and the ‘Fast’ Food pattern, which adds to the findings of studies showing that boys are more likely to have an unhealthy intake pattern [46,47]. In line with previous studies [1,9,48], parental educational level was found to be inversely associated with scores for the Television–Snacking pattern. The Television–Snacking pattern and the Sports–Computer pattern were also positively associated with parental BMI. Interestingly, these two patterns were both also associated with an increased overweight risk for the child at a later age, which could indicate a mediating role of these behavioral patterns in the relationship between parental BMI and children’s BMI, and the intergenerational transmission of overweight and obesity risk. Although previous research assessing individual energy balance-related behaviors as a mediator in the intergenerational transmission of overweight has shown little evidence for such mediation [49], the examination of behavioral patterns as a mediator may provide additional insights in this respect.

All data used in the current study, including those regarding eating routines, activity-related behavior and anthropometric data, were self-reported by the parents, which may have led to bias. Some have suggested the use of accelerometry instead of self-report data in studies examining clustering of activity-related behaviors in children [50]. However, although accelerometer measurements provide objective data on the intensity and duration of activities, they do not distinguish between different activity types [51], and were thus unsuitable for the current study. Although the questionnaire we used to assess activity-related behavior had not yet been validated, previous research has shown that parental reports of BMI are generally quite reliable [52]. However, since BMI does not discriminate between lean mass and fat mass, the association we found with BMI could also reflect associations with lean instead of fat mass. The use of additional measures, such as waist circumference [53], is therefore recommended in future studies using BMI as a measure of childhood overweight. Furthermore, the sampling approach we used meant that families with an ‘alternative’ lifestyle were overrepresented in our study population [29]. Hence, the study population is probably not representative of the general Dutch population, warranting caution when generalizing the results. The children in the current study population had a slightly lower mean BMI than the reference population, for example. On the other hand, all regression analyses were adjusted for the recruitment channel. An additional limitation is the drop-out rate of participants between age 5 and the final follow-up at age 7–8 years, although such drop-out is inevitable in longitudinal studies. Children of mothers with a low educational level and a higher BMI were more likely to drop out, which also limits the generalizability of our findings. Another limitation is that the effect sizes found in the regression analyses were small. A final limitation lies in the analyses. PCA relies on various subjective choices which influence the outcomes, such as the choice of the cut-off point for component loadings. In line with recommendations [35], we used a cut-off point of 0.4, although cut-off points in previous clustering studies were found to range between 0.2 and 0.6. A different cut-off point would have led to different patterns. The findings of the current study are therefore of an indicative nature, and further examination of cross-behavioral clustering of energy balance-related behaviors in children is needed.

## Conclusions

The current study found clustering of energy balance-related behaviors, both within behaviors (i.e. the Sports–Computer pattern including only activity-related behaviors, and the ‘Fast’ Food pattern including only eating patterns) and across behavioral categories (i.e. the Television–Snacking pattern and the Traditional Family pattern). The Television–Snacking pattern and the Sports–Computer pattern were both related to an increased overweight risk at a later age. These findings indicate that future interventions to prevent childhood overweight may profit from addressing eating- and activity-related routines simultaneously, using the synergy between the clustered behaviors. In addition, within behavioral categories (i.e. within eating- and activity-related routines) such interventions should address the wide range of obesogenic behaviors which are important in young children, and not focus on single behaviors. An example of this is that reducing sedentary times seems at least equally important for overweight prevention as increasing physical activity.

In addition, future clustering studies should consider incorporating eating routines instead of, or perhaps in addition to, dietary intake behaviors in their analyses, depending on the research questions. Finally, our results underline the importance of including various activity-related behavior types in clustering analyses, as these behavior subtypes were shown to cluster within different patterns.

## Abbreviations

BMI, Body mass index; KOALA, Dutch acronym for Child, Parent and health: Lifestyle and Genetic constitution; OR, Odds ratio; PA, Physical activity; PCA, Principal component analysis.

## Competing interests

The authors declare that they have no competing interests.

## Authors’ contributions

All authors made substantial contributions to the design of the study. JSG, AS, RAG and CT were involved in the data acquisition. JSG analyzed and interpreted the data, and wrote draft versions of the manuscript. SPJK contributed to the interpretation of the data and the writing of the manuscript. All authors were involved in critically revising the manuscript, and have given their approval to the manuscript as submitted.
